# Editorial: Nutrition and neurodegenerative diseases: insights and perspectives on prevention strategies

**DOI:** 10.3389/fnut.2023.1272338

**Published:** 2023-10-12

**Authors:** Xiaoran Liu, Usune Etxeberria, Miguel Ruiz-Canela

**Affiliations:** ^1^Department of Internal Medicine, Rush University Medical Center, Chicago, IL, United States; ^2^Rush Institute for Healthy Aging, Chicago, IL, United States; ^3^Basque Culinary Center, Faculty of Gastronomic Sciences, Mondragon Unibertsitatea, Donostia-San Sebastián, Spain; ^4^BCC Innovation, Technology Center in Gastronomy, Basque Culinary Center, Donostia-San Sebastián, Spain; ^5^Department of Preventive Medicine and Public Health, University of Navarra, IdiSNA, Pamplona, Spain; ^6^Consorcio Centro de Investigaciones Biomédicas en Red (CIBERObn), Institute of Health Carlos III (ISCIII), Madrid, Spain

**Keywords:** diet, nutrition, prevention, dementia, Alzheimer's disease, environment, inflammation, cognition

Alzheimer's disease (AD) is a growing concern, with 6.7 million Americans aged 65 and older affected in 2023, and this number is projected to double by 2050 (1). Despite decades of research, there are limited disease-modifying therapies, underscoring the importance of primary prevention through diet and lifestyle factors. In fact, the relationships between lifestyle factors and Alzheimer's disease have been an area of active research for several decades. Early observations in the 1990s suggested that nutrients with antioxidant capacities could potentially reduce oxidative stress and inflammation, which are thought to contribute to AD (2, 3). Despite evidence from observational studies and animal studies showing that nutrients with antioxidant and anti-inflammatory capacities delayed the onset of AD and slower cognitive decline (3–6), randomized controlled trials on antioxidant supplementation and cognitive function and AD risk reported mixed results (3–12). It is crucial to consider nutrients within the context of dietary patterns and their sources (dietary vs. supplements), as they are not consumed in isolation. Increasing research has focused on the effects of dietary patterns (7) and nutrients from dietary sources (8) and their relation to brain health (9). Several randomized controlled trials, such as the PREDIMED (10), and more recently the MIND diet trial (11), have demonstrated that healthy dietary patterns prevent cognitive decline. However, most of the current evidence comes from studies conducted in Western countries, highlighting the need for research on diverse dietary patterns globally. Additionally, there is an urgent need to improve our understanding of external factors related to diet quality as well as the effects of multimodal interventions, in line with the FINGER trial (12). In this Research Topic, “*Nutrition and neurodegenerative diseases: insights and perspectives on prevention strategies*,” we included topics that cover environmental and dietary factors associated with cognition.

Wen et al. conducted a systematic review of the effects of pesticides on cognition and brain health, and as well as the potential mechanisms of action based on data from basic research and human studies. Cognitive decline was found to be closely related to oxidative stress, mitochondrial dysfunction, and neuroinflammation induced by pesticide exposure through direct and indirect pathways. The review also highlighted the potential protective effects of natural compounds such as polyphenols, terpenoids, and vitamins on cognition. Furthermore, Liu et al. investigated the association between dietary vitamin E intake and the risk of incident dementia in older Chinese adults. Their analyses, based on data from the Shanghai Aging Study, included 1,550 non-demented community residents aged 60 years and older, with an average follow-up of 5.2 years. They found that higher dietary intakes of vitamin E were significantly associated with a lower risk of incident dementia. Notably, the study examined vitamin E from dietary sources adding to the evidence base that nutrients from different sources (dietary vs. supplementation) may have different relations with cognition.

Nutrients are not consumed in isolation but within a dietary matrix, and a balanced dietary pattern might exert beneficial effects through synergetic effects with other foods and nutrients. In this sense, Rivan et al. assessed the relationship between dietary patterns and the risk of incident mild cognitive impairment and dementia in an East Asian population. The study reported that higher intakes of “tropical fruits-oats” dietary patterns were found to be protective against the risk of dementia incidence among 280 Malaysian older adults during a 5-year follow-up. However, in contrast with previous findings, Rivan et al. observed that higher consumption of the “local snacks-fish and seafoods-high salt foods” was associated with a higher risk of dementia. It is worth noting that the specific types of fish and cooking methods used may contribute to these discrepancies. The preference for deep-fried cooking style among the Malaysian population might hinder the nutrient values of fish consumption. In another study conducted by Ding et al., a meta-analysis explored the association between dietary inflammatory index and global cognitive as well as domain-specific cognitive function. Among the 12 studies in the analysis, a proinflammatory diet was related to a higher risk of developing mild cognitive decline and decline in cognitive domains. These findings are indicative that dietary intervention with anti-inflammatory properties could be beneficial in preserving cognitive health. However, further research is warranted to advance our understanding of the role of an anti-inflammatory diet in brain health.

Accumulating evidence supports the significant role of diet and other lifestyle/environmental factors in preventing cognitive decline and preserving brain health. With an increasing number of studies being conducted globally, these data emphasize the relevance of investigating further on (1) the relationship between diverse dietary patterns from different regions and cognition, and (2) how specific nutrients within the food matrix influence the risk of dementia and AD. Further controlled intervention studies are needed to advance research in this field of nutrition, providing more robust evidence and a deeper understanding of the impact of dietary intervention on brain health.

## Author contributions

XL: Writing—original draft. UE: Writing—review and editing. MR-C: Writing—review and editing.
